# Removal of single and dual ring thiophene’s from dodecane using cavitation based processes

**DOI:** 10.1016/j.ultsonch.2022.106148

**Published:** 2022-08-29

**Authors:** Peter Delaney, Varaha P. Sarvothaman, Ronan Colgan, Sanjay Nagarajan, Gunjan Deshmukh, David Rooney, Peter K.J. Robertson, Vivek V. Ranade

**Affiliations:** a School of Chemistry and Chemical Engineering, Queen’s University Belfast, Belfast BT9 5AG, UK; b Sustainable Environment Research Centre, University of South Wales, Pontypridd CF37 1DL, UK; c Bernal Institute, University of Limerick, Limerick, Ireland

**Keywords:** Oxidative desulphurization, Cavitation, Hydrodynamic, Acoustic, Intensification

## Abstract

Utilising cavitation for enhancing oxidative desulphurization has been investigated for nearly-two decades with recent investigations shifting focus from low-capacity acoustic cavitation (AC) to scalable hydrodynamic cavitation (HC). This work focuses on developing a viable means for removing thiophene’s from fuels. In the first phase of this work, use of vortex based HC devices for removal of single and dual ring thiophenes from dodecane was investigated. HC was shown to be able to remove single ring thiophene from dodecane without using any external catalyst or additives. However, in absence of catalyst or additives, it was not possible to remove dual ring thiophenes such as dibenzothiophene using HC. Therefore, in the second phase of this work, various strategies based on use of catalyst or additives to augment cavitation based process were investigated. AC based experiments were opted for shortlisting suitable catalysts and additives for intensifying cavitation based processes. The influence of using oxidant (H_2_O_2_) and carboxylic acid catalysts on efficacy of removal of dual ring thiophenes is presented. Several conditions were tested, and the optimal volumetric ratios of 0.95 v/v % H_2_O_2_ and 6.25 v/v % HCOOH was identified and utilised throughout the remainder of the study. Regeneration of extractant which accumulates oxidised sulphur species from dodecane was also investigated using AC. The additives and process conditions reported in this work are useful for enhancing desulphurization performance.

## Highlights

• Evaluated removal of dual ring thiophene’s from dodecane using cavitation based processes.
• Hydrodynamic cavitation without additives was unable to remove dual ring thiophene’s.
• Used various additives with cavitation and quantified removal of dual ring thiophene’s.
• The recommended additives are useful for enhancing desulphurization performance.

## Nomenclature

add: Additives
aq: Aqueous phase
C_0_: Initial concentration (ppm)
k: Apparent rate constant (min^−1^)
n: Number of passes through cavitation device (-)
org: Organic phase
T: Temperature (K)
V: Volume (mL)
V_f_: Volume fraction
ΔE/R: Activation energy for pollutant (K)
Δ*P*: Pressure drop across cavitation device (kPa)

**Acronyms**
4,6-DMDBT: 4,6-Dimethyldibenzothiophene
AC: Acoustic Cavitation
ACN: Acetonitrile
ADS: Adsorptive Desulphurization
AOP: Advanced Oxidation Process
BDS: Biodesulphurization
BT: Benzothiophene
CH_3_COOH: Acetic Acid
Cou: Coumarin
DCA: 2,4-Dichloroaniline
DBT: Dibenzothiophene
DBTO: Dibenzothiophene sulfoxide
DBTO_2_: Dibenzothiophene sulfone
DCA: 2,4-Dichloroaniline
EDS: Extractive Desulphurization
H_2_O: Water
H_2_O_2_: Hydrogen Peroxide
HC: Hydrodynamic Cavitation
HCOOH: Formic Acid
HDS: Hydrodesulphurization
HOO•: Perhydroxyl radical
MeOH: Methanol
OH•: Hydroxyl radical
PC: Photocatalysis
PTA: Phase transfer agent
RCOOH: Carboxylic acid
SO_x_: Sulphur oxides
UAOD: Ultrasound Assisted Oxidative Desulphurization

**Greek Symbols**
β: Ratio of flow rate through cavitation device and holding tank volume (s^−1^)
η: Conversion per unit additive volume (%/mL)
θ_add_: Volumetric ratio of additives (-)
∅: Per-pass removal constant (-)
$\emptyset_{0}$: Per-pass removal coefficient (-)
μ: Viscosity (mPa·s)

## 1 Introduction

Irrespective of a global focus on reducing reliance on petroleum and other liquid fuels, international consumption has steadily increased over the past decade. Daily demand for crude oil is expected to exceed pre-pandemic (COVID-19) levels by 2023 as global economy strives for a rapid recovery. Sulphur (S) containing species in crude oil end up in commonly used liquid fuels such as diesel, kerosene and petrol. Combustion of these fuels leads to the emission of sulphur oxide compounds (SO_x_) which can reduce crop yields, contribute to acid rain production, lead to acidification of water bodies and decline the quality of urban air. Attempts to negate the detrimental effects of S emission have taken the form of increasingly rigid legislation implemented by governing bodies worldwide placing stringent regulations for on-road fuels. A sulphur content of 1 ppm in liquid fuels is equivalent to 10 million kilograms of SO_2_ emitted annually. Shipping vessels have also been subjected to stringent regulations with the International Maritime Organisation (IMO) reducing the sulphur concentration from 3.5 wt% to 0.5 wt% on 1st January 2020 [1]. One of the challenges facing the industry is that shipping fuels often are processed at small capacity port refineries or at times on board shipping vessels with scrubbers. The industrial standard, hydrodesulphurization (HDS) requires large volumes to operate in an ecologically and economically sustainable manner and therefore HDS is not quite suitable for small-scale processing facilities. Besides this, some of the S containing compounds such as single and dual ring thiophenes are extremely difficult to remove by HDS because these species sterically hinder the Co-Mo or Ni-Mo catalysts employed in HDS. To remove these refractory compounds, temperatures between 300 and 450 °C and pressures of 3 – 5 MPa are required which consume excessive amounts of hydrogen and catalyst, requiring larger reactors or additional units, which drive up the refinery’s capital expenditure [2]. The high energy consumption of this process restricts refineries from producing fuel with ≤ 1 ppm sulphur which is required to prevent poisoning of catalysts utilised in emerging technologies such as fuel cells, necessitating an alternative approach.

There are limited alternative technologies for HDS, with adsorptive desulphurization (ADS) [3], biodesulphurization (BDS) [4], extractive desulphurization (EDS) [5] and oxidative desulphurization (ODS). ODS being the most commonly investigated, exhibits favourable chemistry compared to HDS as the aromatic sulphur compounds inhibiting HDS, possess high centres of electron density making the sulphur atom susceptible to attack by oxidising species generated in-situ [6]. This complementary relationship may have high potential for reducing energy demands in refinery set-ups or construction of small capacity plants suitable for on-site fuel treatment.

A typical ODS process typically includes an organic and aqueous phases. The aqueous phase contains oxidant and catalyst which react to form species for oxidizing sulphur containing species [7]. The process is limited by mass transfer between the two immiscible liquid phases. Several techniques to overcome and enhance ODS performance have been investigated such as: heterogeneous catalysis [8], [9], aeration [10] and high speed mixing [11] amongst others. Over the past 2 decades, use of cavitation for ODS has been investigated. Of the two methods of cavitation pursued for industrial applications – acoustic cavitation (AC) is energy-intensive and difficult to scale up. Moreover, it has operational problems such as limited penetration depth and the leaching of the probe material etc. The works of Pandit and co-workers discuss that the higher energy requirement and difficulties in scale-up as key issues of AC technology [12]. However, it is suitable for parametric studies with additives as it utilises low processing volumes. Hydrodynamic cavitation (HC) can be scaled up relatively easily and is more energy efficient compared to AC in terms of cavitational yield [13].

Recent publications have focused on using HC systems for oxidative desulphurization by combining water and fuel utilising hydrodynamic cavitation (HC) devices without additives. HC was applied to a range of model fuels (octanol, toluene, octane, diesel) [14] and pyrolysis oil [15]. These studies focus on single ring structure thiophene and an unspecified sulphur pool. In this study, we aim to extend these works to oxidise dual ring aromatic structures (benzothiophene, dibenzothiophene and 4,6-dimethyldibenzothiophene) in dodecane after verifying the effectiveness of HC for removing single ring thiophene. After identifying limitations of HC in removing dual ring thiophenes, we investigated catalysts, oxidants and additives for augmenting cavitation based process. This parametric study was carried out using AC with hydrogen peroxide as an oxidant and carboxylic acid as a catalyst. The study was designed to identify the optimum volume fraction of the aqueous phase (hydrogen peroxide + carboxylic acid) and attempt to reduce additive consumption systematically. A common practice in literature is combining ODS with an extraction or adsorption step where sulphur removal is quantified from a combination of these processes. Throughout the study we have avoided using additional steps for fuel purification, as suggested by Bhasarkar et al. [16]. We specifically quantified oxidation performance as the removal. After completing scoping experiments below, approximately 30 % removal could be achieved using common extractants such as acetonitrile and methanol with a simple washing step. In this work, we have used a modified way of treating extraction solvents in attempt to reduce the energy requirements of the process. The presented results will be useful for developing effective cavitation based ODS process.

## 2 Materials and methodology

### 2.1 Materials

Acetonitrile (ACN) (≥99.9 %, Sigma Aldrich), *n*-dodecane (99 %, Thermo Scientific), *n*-hexane (≥99 %, Sigma Aldrich), Methanol (MeOH) (≥99.8 %, Fisher Scientific) and Toluene (≥99 %, Sigma Aldrich) were utilised for the organic phase throughout this study. The bulk of experiments were completed with *n*-dodecane (C_12_H_26_) for imitating diesel whereas *n*-hexane (C_6_H_14_) was utilised for imitating petrol. Model sulphur species were: 4,6-dimethyldibenzothiophene (DMDBT) (97 %, Thermo-scientific), thiophene (>98 %, Sigma Aldrich), benzothiophene (BT) (98 %, Sigma Aldrich) and dibenzothiophene (DBT) (98 %, Sigma-Aldrich). AC based ODS experiments were carried out with 30 v/v % H_2_O_2_ (Scientific Lab Supplies) as the main oxidant and Acetic acid (CH_3_COOH) (99 %, Sigma Aldrich) or Formic acid (HCOOH) (99 %, Sigma Aldrich) as acid catalysts. Organic pollutants used were 2,4-dichloroaniline (DCA) (98 %, Tokyo Chemical Industry), salicylic acid (SA) (98 %, Sigma-Aldrich) and coumarin (Cou) (98 %, Acros Organics). Acetonitrile was also used as the mobile phase for HPLC-UV-FLD [30].

### 2.2 Experimental set-ups

**Fig. 1:**
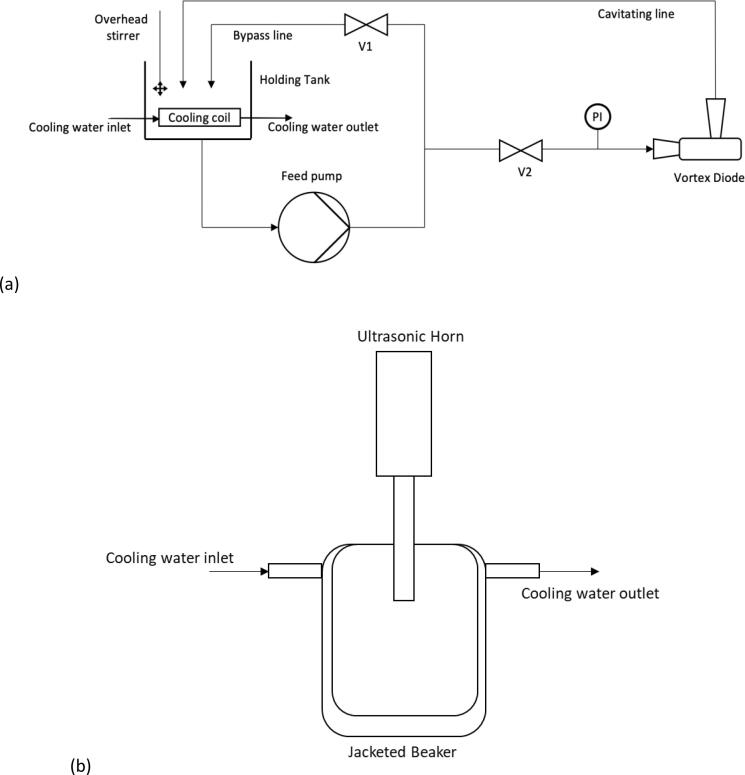
Experimental set-ups. a) Schematic of HC set-up, b) Schematic of AC set-up.

The experimental setup used to carry out HC based ODS in this work comprises of a holding tank/pump/main line/bypass line and a cavitation device. The piping material was first designed using PVC and the pump, as used in our previous study was employed here [17]. Experiments were carried out by adding thiophene to dodecane and mixing with water. When using organic media, the choice of pump and piping material is important. Based on initial scoping experiments, Grundfos CM 1 – 5 self – priming pump (2900 rpm, 50 Hz, single phase), and the piping material comprised majorly of stainless steel and plastic pipes (after checking its compatibility with dodecane) were chosen as compatible pump/material of construction. The HC device used in this work was a stainless steel vortex diode with throat diameter, d_t_ = 6 mm purchased from Vivira Technologies. More details on the geometry and flow characteristics of this device may be found in Simpson and Ranade [18], [19]. Details of experimental set-up and performance of HC device in terms of degradation of organic pollutants may be found in earlier work [19]. The schematic of HC experimental set-up is shown in Fig. 1 a.

In a typical HC experiment, a desired quantity of water and dodecane (model fuel) containing sulphur pollutant was mixed in the cavitation flow loop. Experiments to quantify the effect of pressure drop, volume fraction and initial concentration were conducted with temperature maintained at 28 ± 2 °C, using cooling water circulated through a stainless-steel cooling coil connected to mains water and placed in the holding tank. As for the effect of organic fraction, the precalculated organic and aqueous ratio was mixed by measuring both liquids (water and dodecane). HC ODS has looked at the use of vortex diode based devices (Uebe *et al*.[15]) and also compared the vortex diode with an orifice (Suryawanshi et al. [14]). The studies reporting the use of HC ODS, by the approach of mixing fuel and water have considered either very high, ∼10,000 ppm (Uebe et al. [15]) or very low initial concentrations (up to 300 ppm). In this work, 2500 ppm was selected as the initial concentration which corresponds to approximately 950 ppm of sulphur. Pressure drop is one of the primary device parameters to be investigated for a cavitation based application. In a cavitation device, the inception of cavitation depends on the physicochemical characteristics of the feed (in this case emulsion of dodecane – water), operating temperature and dissolved gases. In a previous work with a similar pump and cavitation device [17] with only water, Sarvothaman reported that the cavitation inception occurs at less than 100 kPa pressure drop [20]. In order to avoid any ambiguity, all the HC experiments in this work were carried out with pressure drop of 200 kPa. These experiments are summarized in Table 1.

**Table 1:** Comparison of parameters investigated in HC system – degradation of DBT and DMDBT.

v_org_ (%)	Aqueous phase pH	T (^o^C)	Thiophene – C_0_ (ppm)	DBT - C_0_ (ppm)	DMDBT - C_0_ (ppm)	Number of passes	Removal of dual ring thiophene from Organic phase
2.5	7	28	2500	x	x	35	NA
10	7	28	x	500	x	500	No
10	7	28	x	100	x	1100	No
10	7	28	x	20	x	500	No
2.5	7	28	x	20	x	500	No
10	7	28	x	x	100	500	No
10	7	45	x	x	100	500	No

For the AC experiments, overall experiments may be grouped into three main categories as described in Table 2. These AC experiments were carried out with dual ring thiophenes as S containing species. Category A focused on identifying the optimum acoustic parameters and baseline additive volumes (A1). Once fixed, the effect of catalyst, water addition and volumetric ratios of additives were examined (A2). After the optimum additive volumetric ratios were identified a set of experiments were completed to systematically reduce these whilst maintaining the H_2_O_2_: HCOOH ratio. Influence of pollutant type and pool of pollutants were also investigated (A3). Category B experiments were aimed to regenerate common extractants (ACN and MeOH) with AC + HCOOH/H_2_O_2_ (B1) and investigate the optimum process parameters (B2). Category C compares stirred conditions for a pool of pollutants in *n*-hexane and *n*-dodecane (C1). For AC experiments a Sonics Vibra Cell 500 W VCX 500 ultrasonic generator was used with ultrasonic horn attachment. A schematic of experimental set-up is shown in Fig. 1 b. The ultrasonic horn tip has a diameter = 13 mm and was submerged 1 cm into solution for each experiment. Unless stated otherwise, the acoustic conditions were set as: frequency = 20 kHz, duty cycle = 50 % (3 s ON/3 s OFF) and acoustic amplitude = 80 %. The vessel utilised was a jacketed beaker with a volume = 50 mL. Temperature was maintained at 22 ± 2 °C by connecting the jacketed beaker to the mains water supply and circulating water through the jacket. Stirred experiments were carried out using a 50 mL glass beaker with magnetic stirrer on a MS-H-Pro+ stirring plate. The stirrer was set at 350 rpm to prevent spilling of the reaction medium. In each experiment the required volume of HCOOH was added first followed by H_2_O_2_ and then the AC power was switched on.

**Table 2:** Summary of experimental conditions and materials.

Experiment Category	Experiment Description	Equipment	Reactor Vessel	Fuel	Pollutant [C_0_ (ppm)]	Oxidant	Catalyst	Analysis
A1	Acoustic Parameters	Sonics VCX 500 Acoustic Horn	50 mL Jacketed beaker	Dodecane	DBT (3 0 0)	H_2_O_2_	HCOOH	HPLC-UV-FLD
A2	Additive Optimisation	Sonics VCX 500 Acoustic Horn	50 mL Jacketed beaker	Dodecane	DBT (300, 600, 1200)	H_2_O_2_, H_2_O	HCOOH, CH_3_COOH	HPLC-UV-FLD
A3	Effect of Pollutants and Additives	Sonics VCX 500 Acoustic Horn	50 mL Jacketed beaker	Dodecane	BT, DBT, DMDBT (300 + 300 + 300)	H_2_O_2_	HCOOH	HPLC-UV-FLD
B1	AC-Extractant regeneration	Sonics VCX 500 Acoustic Horn	50 mL Jacketed beaker	Acetonitrile, Methanol	DBT ( 300)	H_2_O_2_	HCOOH	HPLC-UV-FLD
B2	AC-Extractant regeneration optimisation	Sonics VCX 500 Acoustic Horn	50 mL Jacketed beaker	Acetonitrile, Methanol	DBT ( 300)	H_2_O_2_	HCOOH	HPLC-UV-FLD
C1	Stirring	MS-H-Pro + stirring plate	50 mL Beaker	Dodecane, Hexane	BT, DBT, DMDBT (300 + 300 + 300)	H_2_O_2_	HCOOH	HPLC-UV-FLD

### 2.3 Analytical techniques

For HC based experiments a sample of the emulsion was collected at dedicated time intervals. After approximately 10 min, there was a distinct separation of the aqueous and organic phases. The organic layer was collected and analysed on an Agilent Gas Chromatography (GC) technique equipped with an FID detector (initial temperature = 60 °C, ramp rate = 20 °C/min and final temperature = 250 °C). As experiments included low organic volume fractions, in those experiments – a sample volume of 250 mL was collected into a standard flask, the ‘organic’ layer was centrifuged at 15,000 rpm for 15 min, after ensuring that the organic layer was free of any water by visual inspection.

For all AC experiments, samples were taken at regular intervals by stopping the experimental equipment at regular intervals (10, 20 and 30 min), (the acoustic horn contained in-built time relay) and pipetting 2 mL from the reaction vessel into a centrifuge tube. Phase separation occurred almost instantaneously, and the volume of the aqueous phase was negligible. The initial sample for t = 0 min was taken from the stock solution. After the separation step 1 mL of sample was transferred to a HPLC vial and analysed using High performance liquid chromatography – UV – fluorescence detection (HPLC-UV-FLD). A Kinetex 5 µm C18 column was used for this purpose. Mobile phases of solvent A – pure acetonitrile and solvent B – deionised water was prepared to mobilise the samples through the stationary phase. A linear flow gradient from 100 % A, 0 % B (run-time = 0 min) to 90 % A, 10 % B (run-time = 8 min) gave the most effective separation between components. No additional run-time was provided. A sample volume of 5 μL was injected into the column using an autosampler. The samples were analysed using a PDA-100 Photodiode Array Detector. The UV detector was set at a wavelength of 287 nm and the mobile phase flow rate was 0.5 mL/min. Categories B1 and B2 are single phase as acetonitrile/methanol are miscible with the polar phase and were simply collected and analysed using HPLC-UV-FLD without centrifugation. The analytical technique accuracy for both the GC and HPLC techniques was within ± 1 %, this was calculated by testing identical samples multiple times during the calibration and real experimental runs.

### 2.4 Estimation of per-pass removal and kinetic modelling

To accurately compare the degradation in HC set-ups with other relevant publications a per – pass modelling approach was developed in our previous work for estimating the degradation performance of organic pollutants in water [17] was used. The overall behaviour of such a set-up can be modelled as:

(1) $$V\frac{\mathrm{dC}}{\mathrm{dt}}=q\left(C_{\mathrm{in}}-C\right)-Q\emptyset C$$

Here C is the concentration of sulphur species in the organic phase (and emulsion), V is the volume of entire holding tank (aqueous and organic phase), Q is a flow rate through cavitation device and ∅ is a per – pass removal. If the value of ∅ is assumed to be constant over a range of concentration time, this value can be estimated by running the cavitation set-up in a batch mode and using experimental measurement of concentration of pollutant as a function of time and Eq. (1) with q = 0 as:

(2) $$C=C_{\mathrm{in}}e^{-\beta \emptyset t}$$

where β is a ratio of flow rate through cavitation device and holding tank volume (Q/V, s^−1^). The product of β and time indicates number of passes, n, through the cavitation device for a batch system:

(3) n=βt

The value of per-pass degradation factor, ∅ may be obtained by fitting the experimental data using Eq. (2).

For batch AC set-ups, the apparent rate constant was assumed to follow pseudo-first order kinetics and calculated using Eq. (4):

(4) $$C=C_{\mathrm{in}}e^{-kt}$$

where k = apparent first order kinetic rate constant (min^−1^).

## 3 Results and discussion

The first section presents results with HC for removing sulphur containing species in the organic phase, dodecane. Experiments were initially conducted with thiophene as model S containing species since it is most recalcitrant for ODS to treat at ambient conditions and to reconfirm previously published reports. Experiments were carried out with vortex based cavitation device operated with pressure drop (ΔP) of 200 kPa (flowrate of device = 4.2 LPM). Energy dissipation calculations from the HC device can be calculated with data of ΔP and flowrate, by procedure demonstrated by prior studies (Agarkoti et al. [21]). Initial concentration, C_0_ as 2500 ppm and organic volume fraction as 2.5 v/v %. These conditions were selected as the baseline conditions and would be optimised in further studies. Same operating conditions were then extended to dual ring aromatic species in system without any external catalysts or additives.

### 3.1 Hydrodynamic cavitation for single and dual ring thiophene removal

**Fig. 2:**
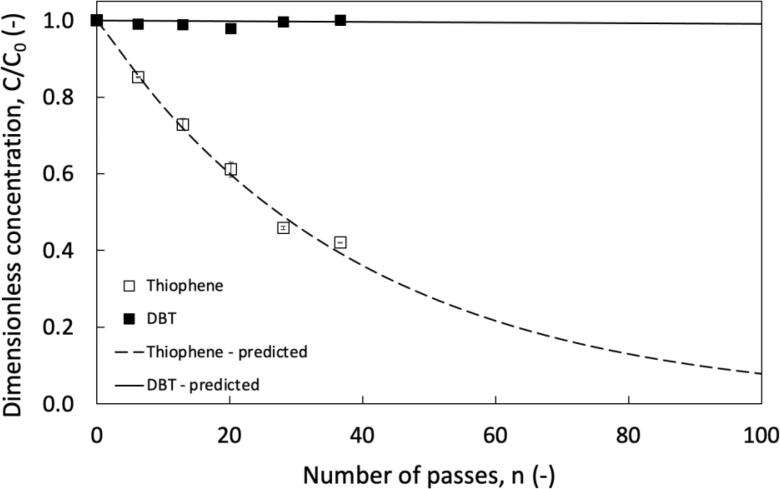
Influence of pollutant type on removal dodecane, v_org_ = 2.5 %, T = 28 ± 2 °C, C_0_ = 2500 ppm, ΔP = 200 kPa, ∅ × 10^3^ for predicted curves = 32.2 and 0.1 for thiophene and DBT respectively; predicted using Eq. (2).

The observed removal of thiophene as a function of number of passes is shown in Fig. 2. It can be seen that the HC vortex based device can substantially remove thiophene from dodecane without any external catalyst or additives. This is in line with the previous studies of Suryawanshi et al. [14]. The dashed line shown in Fig. 2 is based on Eq. (2) with per-pass factor of 0.032. It can be seen that per-pass model is able to describe the experimental data quite well.

Encouraged by these results, further experiments on removal of dual ring thiophenes were carried out in the same manner as thiophene based studies. DBT and DMDBT were selected as model dual ring S containing species. These experiments were also carried out with ΔP = 200 kPa and 2.5 v/v % organic volume fraction. The experimental results for DBT are compared with that obtained for thiophene in Fig. 2. The results obtained with DMDBT were very similar to that observed for DBT and are not shown in Fig. 2 to avoid clutter. It can be seen that almost no degradation of dual ring thiophenes was seen. This suggests that thiophene removal may follow a different degradation mechanism than as oxidation of dual ring thiophenes [6]. In order to further investigate possible removal of dual ring thiophenes, systematic experiments by varying volume fraction of organic phase, pH, temperature, and initial concentration were carried out. These experiments and observed degradation are summarised in Table 1.

Despite covering a wide parameter space (Table 1), removal of dual ring thiophene’s was not possible via HC without any external catalysts and additives. As a reference case, simple extraction experiments were carried out using acetonitrile (ACN). With ACN to dodecane ratio as 3 as to 1 was found to remove 30 % DBT from dodecane. However, HC was not able to remove DBT without external catalysts or additives. To the author’s knowledge, there is no other study which has reported this observation and is often an implicit information. Almost all the studies on removal of dual ring thiophene’s with cavitation use external catalysts and additives to show removal of DBT (Baradaran and Sadgehi [22], [23]; Rajendran et al. [24]). Based on the experimental results obtained in this work and analysis of previous studies, it was decided to investigate possibility of using external catalysts and additives to augment HC based removal of dual ring thiophenes. Typical HC experiment requires working volume of 10–15 L. Systematic experiments to explore different catalysts and additives would therefore require significant volume of chemicals. For minimising the consumption of chemicals and reducing waste generation, it was decided to use acoustic cavitation for identifying promising catalysts and additives. The promising catalysts and additives are identified using AC based small-scale experiments can then be used with HC for removing dual ring thiophenes. The experimental results of removal of dual ring thiophenes using AC and external catalysts/ additives are discussed in Section 3.2. The scope of this work is restricted to identifying suitable catalysts/additives along with potential regeneration of extractant. Results on use of identified catalysts and additives with HC will be presented separately.

It is important to understand the difference between AC and HC prior to switching between these two modes of cavitation: the key differences in AC and HC arise due to the different characteristic pressure fluctuations (in terms of frequency and amplitude). Due to this, the intensity of cavity collapse is quite different. Despite these differences, main characteristics of cavity collapse and resulting generation of intense shear and oxidising radicals are not qualitatively different. Recently Pandit et al. [25] have used detailed cavity dynamics models to quantify influence of pressure fluctuations and frequency on cavitation effects spanning the range used for AC and HC. It can be seen from their results that effective shear and oxidising radicals for the two can be comparable under certain conditions. Several studies have observed similar performance of AC and HC with and without additives for water treatment (see for example, Agarkoti et al. [21]). We therefore believe that the results obtained with AC can be used and transferred judiciously to HC if the changes in pressure fluctuations (frequency and amplitude) as well as extent of cavitation (number density of cavities per unit volume) are accounted for. AC based experiments to identify promising additives are discussed in the following.

### 3.2 Removal of dual ring thiophene’s with external catalysts or additives

Several different catalysts and additives have been used for enhancing removal of sulphur species via ODS (Rajendran et al. [24]; Avvaru et al. [26]). The mechanism for water-based cavitation as proposed by Suryawanshi et al. suggests sulphur oxidation through hydroxylation [13], [14]. Hydroxyl radicals (OH•) may possess too short a life span (10^−9^ s) [27] to significantly oxidise dual ring sulphur species in a heterogeneous set-up (*i.e*. radical lifespan < OH• diffusion timescale and reaction with sulphur atoms). One of the frequently reported systems for ODS is the combination of H_2_O_2_ as an external oxidant and carboxylic acid as a catalyst. The proposed mechanism for removal of DBT with this system is shown by Eqs. (5), (6). H_2_O_2_ and carboxylic acid react to form an unstable intermediate, which rapidly dehydrates forming a peroxycarboxylic acid. Peroxycarboxylic acid reacts with sulphur species to form a sulfoxide which rapidly reacts to form the corresponding sulfones [7], [28]. The carboxylic acid is then regenerated:

(5) $$H_{2}O_{2}+RCOOH\to RCOOOH+H_{2}O$$

(6) $$\mathrm{HCOOOHH}+DBT\to DBTO+RCOOOH\to DBTO_{2}+RCOOH$$

Stoichiometry suggests that one molecule of H_2_O_2_ is required per molecule HCOOH. However, usually excess H_2_O_2_ is required to compensate for side reactions forming by-products and decomposition. H_2_O_2_ consumption may be a limiting factor for oxidation reactions and forms water which dilutes the reaction media [29]. A possible side reaction is the reaction of H_2_O_2_ to form OH• and there is some discussion whether this is beneficial or detrimental for oxidation. Several authors suggest it aids oxidation of sulphur species [30], [31], [32] whilst others comment that it may act as a scavenger of reactive perhydroxyl radicals (HOO•) produced from the intermediate acid species [28], [29], [33], [34] or lead to acid decarboxylation of carboxylic acid [35]. Our HC work displaying lack of hydroxylation for dual ring structures suggests that the role of cavitation is primarily physical which is also reported by several AC based publications [29], [36]. To examine the effect of cavitation in an additive based ODS system, we selected H_2_O_2_ and HCOOH as oxidant and catalyst/oxidant promoter respectively. It was necessary to determine appropriate parameters of AC system at fixed volumes of H_2_O_2_ and HCOOH to ensure the formation of a fine emulsion and a well-mixed system.

To evaluate the additive-based system, preliminary experiments varying the volume ratios of H_2_O_2_ and HCOOH were set up. Our baseline conditions were selected as 2.5 mL formic acid and 1.5 mL 30 v/v % H_2_O_2_. Acoustic conditions were determined by altering the amplitude as 40, 60 and 80 %. 40 and 60 % amplitude showed two distinct phases with the denser aqueous phase settling at the bottom of the reactor vessel. 80 % amplitude was selected as excellent dispersion of the aqueous phase was achieved. Duty cycle was also investigated, which is pulsing of the ultrasonic waves (*i.e*. a duty cycle of 50 % is equal periods of the sonicator in ON and OFF positions). Utilization of duty cycle is an effective measure to reduce energy consumption by reducing the total sonication time. To overcome mass transfer limitations a fine emulsion produced by cavitation is required. While selecting duty cycle, it is important to ensure that the emulsion stability is retained for longer than the off cycle [8]. Careful control of the duty cycle can also enhance the rate of reaction, where the optimum rate constant may not necessarily be obtained at higher values. Duty cycles of 3 s ON/3 s OFF, 3 s ON/2 s OFF, 3 s ON/1 s OFF and 5 s ON/3 s OFF, were tested at 80 % amplitude and DBT oxidation of 59.3 ± 3.4 % was achieved. Based on these results acoustic conditions were set at amplitude = 80 % and duty cycle = 50 % (3 s ON/3 s OFF) for the remainder of AC based experiments. The role of cavitation on chemical transformations and additive optimisation was then assessed in a well-mixed system.

**Fig. 3:**
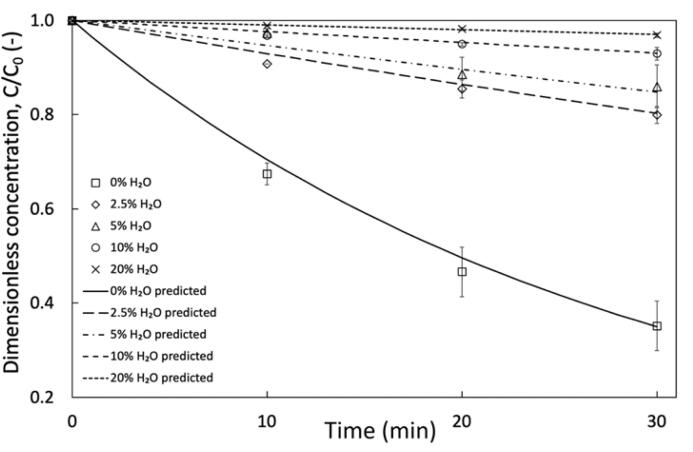
Influence of water addition for DBT oxidation: Reduction of optimised additive dosage for pool of pollutants. Process conditions: Organic phase = dodecane, C_0, BT_ = 300 ppm, C_0, DBT_ = 300 ppm C_0, DMDBT_ = 300 ppm, Acoustic amplitude = 80 %, Duty cycle = 50 % (3 *s* ON/3 *s* OFF), k_0%_ = 0.035 min^−1^, k_2.5%_ = 0.007 min^−1^_,_ k_5%_ = 0.006 min^−1^_,_*k*_10%_ = 0.002 min^−1^_,_ k_20%_ = 0.001 min^−1^.

To evaluate cavitation’s influence on chemical transformations in systems with additives we deliberately added water to see its influence on oxidation and co-extraction. Although water alone cannot oxidise dual ring thiophenes, it may assist the H_2_O_2_/HCOOH system by increasing OH• yield for oxidation or assisting with simultaneous extraction. Experiments were completed by intentionally increasing the aqueous volume fraction [aqueous volume fraction= (volume of HCOOH + H_2_O_2_ + water)/ Total Volume], with water present in the 30 wt% H_2_O_2_ being recorded as the H_2_O_2_ volume. Results shown in Fig. 3 display an inhibitory effect of water as water addition reduces DBT oxidation. Addition of only 1 mL of water (2.5 % of set-up volume) in the system with 2.5 mL HCOOH and 1.5 mL H_2_O_2_, decreases oxidation to 20 % from 64.8 % in absence of externally added water. Addition of water appears to be diluting the aqueous phase and H_2_O_2_ concentration [30], as well as increasing the pH of the medium and restricting formation of peroxyformic acid. Additionally increasing the volume of the aqueous phase may hinder interfacial transfer of S and oxidising species. Similar data was reported by Bhasarkar et al. [19] who investigated water addition in a H_2_O_2_/CH_3_COOH system for different pollutants and concentrations, reporting a slight decline in oxidation of BT, 3-methyl thiophene and thiophene at 100 ppm, 300 ppm and 500 ppm compared to the system without water. Therefore, only H_2_O_2_ and carboxylic acid was utilised hence forth.

**Fig. 4:**
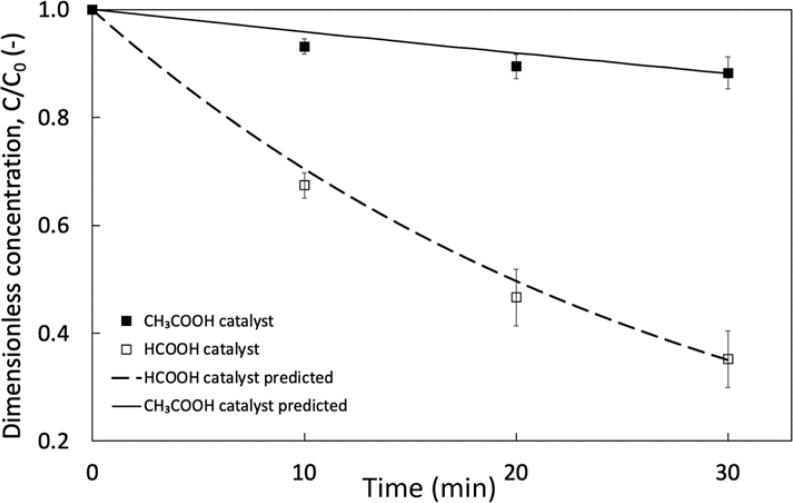
Influence of acid catalyst type for DBT oxidation. Process conditions. Organic phase = dodecane, v_f, org_ = 90 %, v_f, H₂O₂_ = 3.75 %, v_f, acid_ = 6.25 %, C_0, DBT_ = 300 ppm, Acoustic amplitude = 80 %, Duty cycle = 50 % (3 *s* ON/3 *s* OFF), k_HCOOH_ = 0.035 min^−1^, k_CH3COOH_ = 0.004 min^−1^.

Selection of carboxylic acid was investigated to evaluate if there is an impact on DBT oxidation. Results for HCOOH and CH_3_COOH are compared in Fig. 4 at baseline conditions (1.5 mL H_2_O_2_, 2.5 mL acid) achieving 68.5 % and 11 % DBT oxidation respectively. The oxidation achieved for CH_3_COOH is lower than typically reported in literature. Mello et al. compared HCOOH, CH_3_COOH and H_3_CCH_2_COOH achieving 96.7 %, 95.6 % and 77.5 % sulphur removal after 3 stage extraction with MeOH [37]. Although HCOOH performs best, they selected CH_3_COOH as it is more stable and less corrosive. Contrary to this, Bal and Bhasarkar comment that the peroxyformic acid has a higher reactivity towards the organic acid interface [38]. They also add that the smaller molecule has a higher diffusivity for a system including a phase transfer agent (PTA). Their discussion suggests that diffusivity of a PTA-anion complex in a system with *n*-decane is lower than that of toluene from their previous work, of which the *n*-decane system achieved lower oxidation [16]. They hypothesise that this may be a result of the higher viscosity (μ) of the organic phase (*n*-decane, μ = 0.85 mPa·s @ 25 °C, toluene, μ = 0.56 mPa·s). Although the mass transfer mechanism within this work depends on acoustic emulsification rather than ion exchange through a central-complex, the viscosity of *n*-dodecane (μ = 1.34 mPa·s) is larger than *n*-decane. Thus, a lower diffusivity of CH_3_COOH/peroxyacetic acid into the organic layer could provide an explanation for the low DBT oxidation when compared to HCOOH/peroxyformic acid. As higher DBT oxidation was achieved with HCOOH, it was selected as the acid catalyst.

**Fig. 5:**
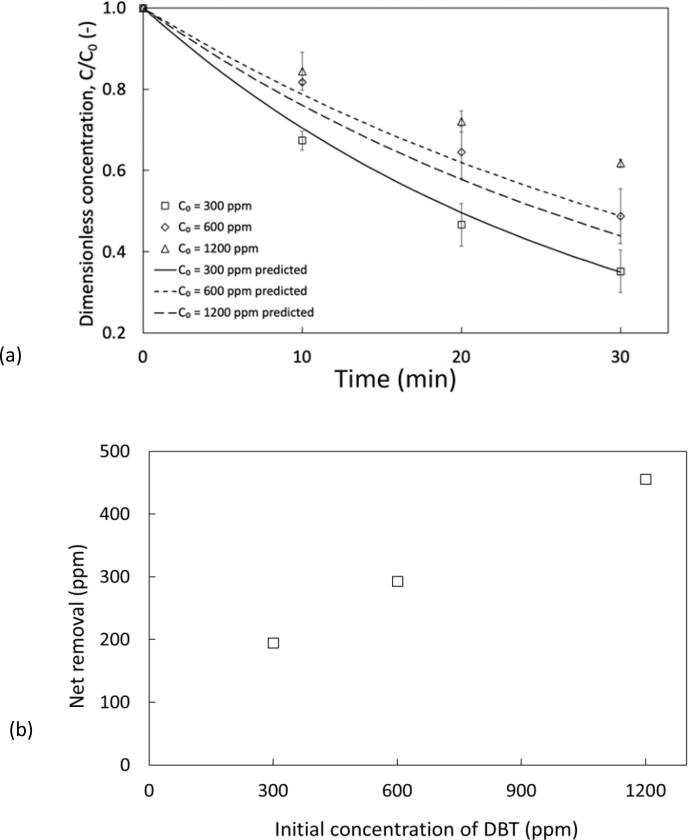
Influence of initial DBT concentration: (a) Organic phase = dodecane, v_f, org_ = 90 %, v_f, H₂O₂_ = 3.75 %, v_acid_ = 6.25 %, Acoustic amplitude = 80 %, Duty cycle = 50 % (3 s ON/3 s OFF), k_C₀_ = _300 ppm_ = 0.035 min^−1^, k_C₀_ = _600 ppm_ = 0.024 min^−1^, k_C₀_ = _1200 ppm_ = 0.027 min^−1^ (b) on net removal

After establishing the optimum ultrasonic parameters and additive selection, we assessed the initial concentration (C_0_) to determine how effective the baseline parameters are when more pollutant is present in the system. Influence of initial concentration is shown in Fig. 5 a. These results show that increasing initial concentration reduces the extent of oxidation, which may be expected as there is a finite amount of oxidant present in the reaction medium. C_0_ = 300 ppm achieved 64.8 % DBT oxidation, whereas 600 ppm and 1200 ppm generate 51.2 % and 38.2 % respectively. However, higher C_0_ values corresponds to higher S removal per unit additives, where the systems containing 600 ppm and 1200 ppm improved DBT oxidation (%) by factors of 1.5 and 2.4 compared to the experiments with 300 ppm, this is plotted as Fig. 5 b. The oxidation extent for varying initial concentration may be based on additive quantities as other authors report opposite trends to this work. Margeta et al. compared the initial concentrations of DBT ranging from 1221 ppm to 3777 ppm in a H_2_O_2_/CH_3_COOH system with heptane, *n*-dodecane and *n*-hexadecane as model fuel. 3777 ppm achieved the best DBT oxidation of 87 % (without an extraction step) and oxidation decreased as concentration was lowered [39]. The influence of initial concentration may be dependent on the system as Bolla et al. 2012 recorded a 1.6 % decline in BT conversion when increasing the concentration from 100 to 500 ppm for a system with H_2_O_2_/CH_3_COOH and toluene as model fuel (44.5 % at C_0_ = 100 ppm vs 43.9 % at C_0_ = 500 ppm) [36]. This suggests that S oxidation could be dictated by the quantity of oxidant, as it is consumed, while the acid catalyst is regenerated. The influence of pollutant should be assessed before optimising additive consumption.

The reactivity of pollutants in ODS is reportedly based on their electron density, for example the three pollutants examined in this work exhibit electron density’s at: BT = 5.739, DBT = 5.758 and DMDBT = 5.760 [6]. Thus, the conversion of DBT and DMDBT to their corresponding sulfones should be higher than BT and smaller dual ring aromatics. This trend is confirmed by our results, as DBT and DMDBT achieved 64.8 % and 57.3 % conversion respectively whereas 22.2 % was obtained for BT. This trend is agreeable with the majority of literature comparing different pollutants with similar trends for AC based systems with PTAs [40], [41], ionic liquids [42] and heterogeneous catalysts [43]. To attempt to realistically assess real world applications, a pool of pollutants should be utilised to mimic the S composition of transport fuels.

Applying the baseline conditions to a pollutant pool containing 300 ppm of each pollutant achieving individual pollutant oxidation of 39.2 %, 76.5 % and 76.2 % for BT, DBT and DMDBT and overall S conversion of 64 %. Similar experiments were conducted by Duarte et al. with H_2_O_2_/CH_3_COOH and toluene, comparing BT (C_0,S_ = 285 ppm, S removal = 64.8 %), DBT (C_0,S_ = 208 ppm, S removal = 96.3 %) and DMDBT (C_0,S_ = 180 ppm, S removal = 98.6 %) after extraction with MeOH [44]. The same study compares pollutant pools where removal decreases with overall initial concentration and the highest of 85.8 % is achieved for the pool which contains the lowest amount of BT and lowest overall S concentration. These trends show that the pool of pollutants can achieve similar oxidation levels as individual pollutants at the baseline conditions, which is unexpected as there is more competition between compounds for the reactive species generated in-situ. This may be a result of the volumetric ratios of additives to S in the study. To maximise S oxidation, a study to identify the optimum volume ratio of HCOOH to H_2_O_2_ was attempted.

**Fig. 6:**
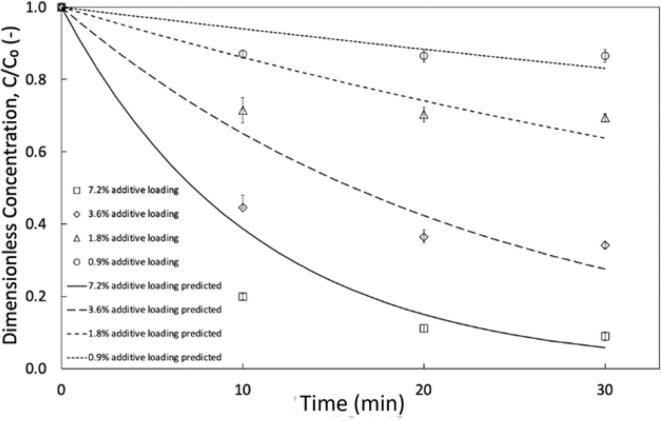
Reduction of optimised additive dosage for pool of pollutants: Process conditions. Organic phase = dodecane, C_0, BT_ = 300 ppm, C_0, DBT_ = 300 ppm C_0, DMDBT_ = 300 ppm, Acoustic amplitude = 80 %, Duty cycle = 50 % (3 *sec* ON/3 *sec* OFF), k_vf,aq_ = _7.2%_ = 0.095 min^−1^, k_vf,aq_ = _3.6%_ = 0.043 min^−1^, k_vf,aq_ = _1.8%_ = 0.015 min^−1^, k_vf,aq_ = _0.9%_ = 0.0062 min^−1^.

To establish the optimum ratios of additives, experiments were conducted by fixing the volume of H_2_O_2_ or HCOOH and modifying the other. In these experiments, the volumetric additive ratios (θ_add_ = V_HCOOH_: V_H2O2_) ranged from 0.67 to 6.67. The highest conversion was achieved at θ_add_ = 3.33 achieving 98.6 % DBT oxidation. However, in this study when assessing the S conversion per volume of additives consumed, we find that the θ_add_ = 6.67 achieves 90.4 % conversion and the most efficient conversion per volume additives (η = X/V_add_ (%_DBT_/mL_add_)). This shows that the θ_add_ = 6.67 was equivalent to 31.4 %_DBT_/mL_add_ whereas θ_add_ = 3.33 set at 16.2 %_DBT_/mL_add_. Consequently, the optimum additives volumes were fixed at V_H2O2_ = 0.375 mL and V_HCOOH_ = 2.5 mL equivalent of a total aqueous volume fraction (V_f, aq_) = 7.20 %, with a composition of 0.95 v/v % H_2_O_2_ and 6.25 v/v % HCOOH. Experiments attempting to reduce additive consumption were then completed by fixing θ_add_ = 6.67 and reducing the additive dosage. These experiments were carried out using the pool of pollutants to mimic actual fuels and were also completed with baseline conditions (θ_add_ = 1.67), a n—hexane medium and in a stirred set-up (stirring was completed for *n*-hexane and dodecane as the organic phase). Results are plotted in Fig. 6 which show a decline in overall S oxidation as the dosage is reduced for all tested conditions. This may be due to the rapid consumption of H_2_O_2_, limiting oxidation at lower dosages.

In the successive section we use the AC-ODS system, to remove sulphur compounds from methanol/acetonitrile, to quantify the utilization of additives in a homogenous media (Section 3.3).

### 3.3 Dosing of additives in a homogenous media: Regeneration of extractant

**Fig. 7:**
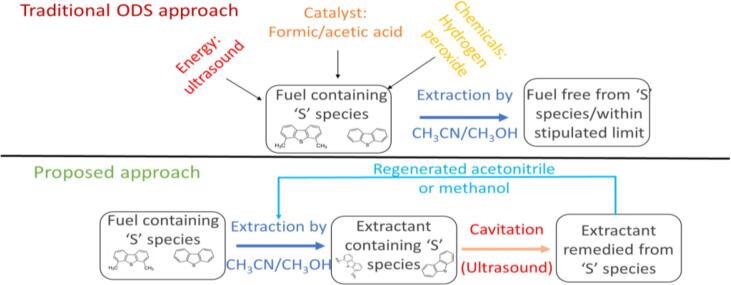
Proposed approach for treating extractant with oxidant/catalyst and AC.

Throughout the study, we have avoided the utilisation of extractants to solely evaluate the oxidation performance of cavitation. It is well documented that washing fuels with an extractant can achieve up to 30–40 % removal [37]. However, this simply transfers the sulphur from one organic phase to another. It is essential to remove S containing species from extractant in order to regenerate and reuse it. We have evaluated use of the optimal HCOOH/H_2_O_2_ system identified in this work for regenerating extractants. In Fig. 7 the approach for this method of dosing the additives is summarized. The proposed approach would depend on extracting unoxidized sulphur species (DBT, DMDBT and so on) from the fuel to the extractant phase. After this, the extractant can be dosed with additives and regenerated. The expected advantage is that additives which are in a homogeneous phase might require smaller dosing and we attempt to quantify potential benefits.

**Fig. 8:**
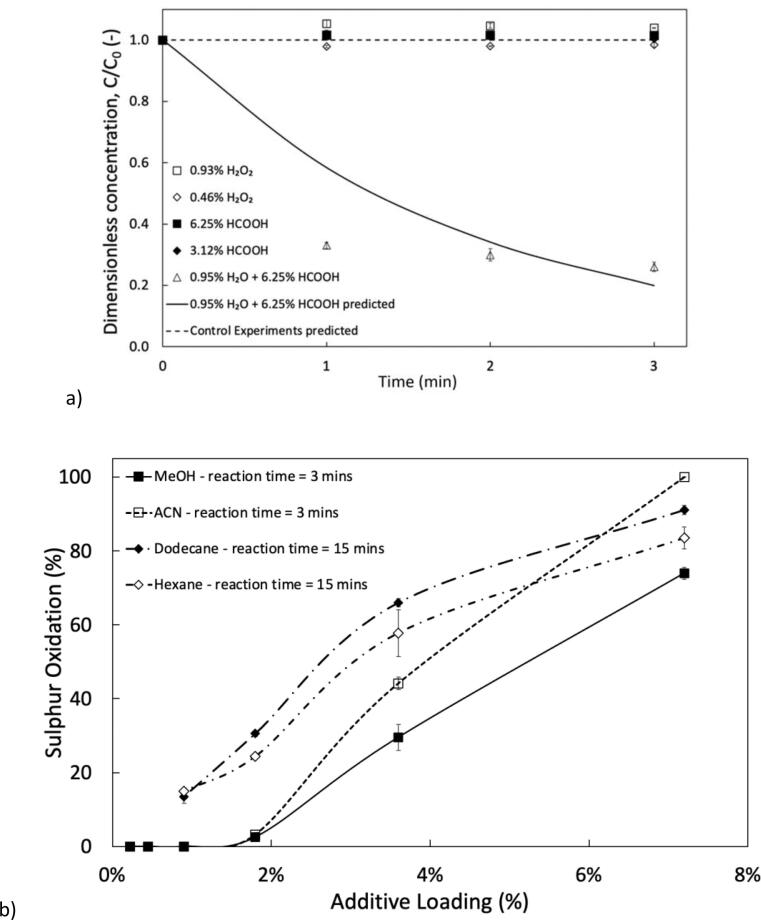
a) Comparison of control experiments with only H_2_O_2_ or HCOOH and optimised conditions (v_f, aq_ = 7.20 %, v_f, H₂O₂_ = 0.95 %, v_acid_ = 6.25 %, k = 0.538 min^−1^) b) reduction of total additives (H_2_O_2_ + HCOOH). Process conditions: Polar phase = MeOH, C_0, DBT_ = 300 ppm, Acoustic amplitude = 80 %, Duty cycle = 50 % (3 s ON/3 s OFF).

Treatment of sulphur in organic solvents such as ACN and MeOH is simpler than fuels as they are miscible with the aqueous phase reducing the mass transfer limitation significantly. Initially, (Fig. 8 a) control experiments were conducted to determine whether H_2_O_2_ or HCOOH alone could effectively remove sulphur. Under these conditions no oxidation was produced which is agreeable with literature comparing these conditions for model fuels. When utilising the optimum ratios of 0.95 v/v % H_2_O_2_ and 6.25 v/v % HCOOH identified earlier in the study 100 % oxidation of DBT could be achieved in under 3 min for ACN and 72.4 % for MeOH. Systematic reduction of the additive volumes was then examined (Fig. 8 b), after reducing the quantity of additives by a factor of 2, DBT oxidation in ACN reduced to 42.5 %. This suggests that the identified volume ratio of oxidant and catalyst the minimum volume required for the system. This process may offer an energy efficient alternative for treating fuels, as the treatment time is lowered by 10 times. There is potential for scalability of this homogeneous system in HC to act in the same role as the AC set-up, with the role of reducing residence times and conserving energy.

**Fig. 9:**
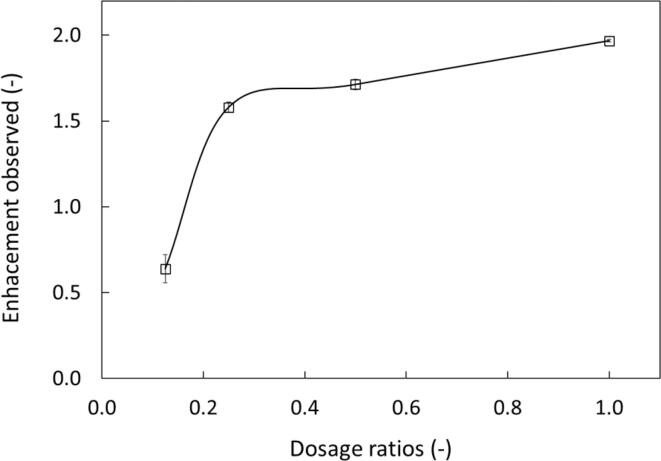
Influence of dosage ratios on the enhancement observed for sonication against stirring. Process conditions. Organic phase = dodecane, C_0, BT_ = 300 ppm, C_0, DBT_ = 300 ppm C_0, DMDBT_ = 300 ppm, Amplitude = 80 %, Duty cycle = 50 % (3 s ON/3 s OFF) and stirring at 500 rpm.

As the next step, we quantified the influence of sonication (A3) over stirring (C1) system. For this, we considered an identical operating volume and pool of pollutants in Table 2, across four different additive dosages. Fig. 9 shows the enhancement factor per additive dosage ratio where the dosage ratio is defined as the volume of additives relative to the maximum volume of additives (V_add_/ V_add, max_) where the maximum volume of additives is the optimised amount identified in this study. The results show that the enhancement is not pronounced at the lowest additive dosage of 0.125, however for the higher additive dosages (0.25, 0.5, 1.0), an enhancement factor of 1.5 and above was obtained. Thus, the incorporation of sonication clearly improves process performance.

We believe the presented results/the systematic investigation of sonication and operating parameters will be of use to further work aimed at desulphurization of liquid fuels.

## 4 Conclusions

The focus of this study was to quantify oxidation of single and dual ring thiophenic species in model fuels using cavitation based ODS. In the first part, use of hydrodynamic cavitation for removing single and dual ring thiophenes from dodecane was investigated. In the second part, investigations were carried out to identify suitable catalysts and additives for enhancing removal of S species. This second part of investigations was carried out using acoustic cavitation to minimize waste generated. Different additive based dosing methods (H_2_O_2_ + carboxylic acid and use of dosing on extractant media) were systematically investigated for the removal of dual ring thiophene species. The key conclusions from the study are as follows:

• Hydrodynamic cavitation can oxidise and remove single ring thiophene from dodecane without any external catalysts or additives. It was however not possible to oxidise dual ring thiophene (BT/DBT/DMDBT) in absence of external catalysts or additives
• Formic acid was found to be superior (65 % removal) to acetic acid (12 % removal) as a catalyst
• Additive dosing of 0.95 v/v % H_2_O_2_ and 6.25 v/v % HCOOH was found to be optimal and effected > 90 % removal
• The process time was reduced by 10 times whilst removing sulphur from acetonitrile (simulated extractant), as compared to sulphur from dodecane (simulated fuel)

The results presented here will be useful to design processes for removing dual ring thiophenes from liquid fuels.

## CRediT authorship contribution statement

**Peter Delaney:** Investigation, Data curation, Validation, Writing – original draft. **Varaha P. Sarvothaman:** Investigation, Methodology, Data curation, Validation, Writing – review & editing. **Ronan Colgan:** Investigation, Validation. **Sanjay Nagarajan:** Methodology, Validation. **Gunjan Deshmukh:** Validation. **David Rooney:** Supervision. **Peter K.J. Robertson:** Supervision, Writing – review & editing. **Vivek V. Ranade:** Conceptualization, Funding acquisition, Supervision, Writing – review & editing.

## Declaration of Competing Interest

The authors declare the following financial interests/personal relationships which may be considered as potential competing interests: Vivek V. Ranade reports a relationship with VIVIRA Process Technologies Pvt. ltd. that includes: board membership.

## Data Availability

Data will be made available on request.
